# Cyanosis Caused by Acetanilid

**Published:** 1888-05

**Authors:** 


### CYANOSIS CAUSED BY ACETANILID

This condition, while not as frequent as the writings of Germain See would indicate, is yet sufficiently common for us always to warn our patients, that they may not be unnecessarily disturbed by such an alarming symptom.

The drug was recently prescribed by the writer for a lady living in an adjoining State. At first it caused no marked symptoms and relieved the hadache for which it was given. One morning, however, after taking two powders, of five grains each, she noticed that her lips were purple; later, her ears, fingers, and face turned the same color. The lady was thrown into the utmost distress, and alarmed her friends. She had suffered from a mild attack of hemiplegia, and these untoward symptoms were looked upon as the sure precursors of death.

A physician recently related an amusing case, to the physician at least, occurring in his practice. He had attended a lady in confinement, the child dying a few days later from general cyanosis. Shortly afterward he prescribed acetanilid for the mother. The following night he was hastily summoned to her house and informed that she was dying from the same disease, which she had *caught* from her child.

This idiosyncrasy is certainly very variable. It is more frequent where large doses are given, but many persons are affected by a small quantity. Again, some patients take the drug for some time before cyanosis develops. The writer has given the drug for weeks, in one instance for five months, without the appearance of this symptom.

So far as our knowledge extends, the condition is free from danger, and does not contra-indicate the use of the drug where the patients do not object. The cyanosis promptly disappears after the withdrawal of the remedy.

